# Pleomorphism and Viability of the Lyme Disease Pathogen *Borrelia burgdorferi* Exposed to Physiological Stress Conditions: A Correlative Cryo-Fluorescence and Cryo-Scanning Electron Microscopy Study

**DOI:** 10.3389/fmicb.2017.00596

**Published:** 2017-04-11

**Authors:** Marie Vancová, Nataliia Rudenko, Jiří Vaněček, Maryna Golovchenko, Martin Strnad, Ryan O. M. Rego, Lucie Tichá, Libor Grubhoffer, Jana Nebesářová

**Affiliations:** ^1^Biology Centre CAS, Institute of ParasitologyČeské Budějovice, Czechia; ^2^Faculty of Science, University of South BohemiaČeské Budějovice, Czechia; ^3^Faculty of Science, Charles University in PragueCzechia

**Keywords:** cryo-fluorescence, cryo-scanning electron microscopy, *Borrelia burgdorferi*, Lyme disease, round body, pleomorphism, viability staining

## Abstract

To understand the response of the Lyme disease spirochete *Borrelia burgdorferi* exposed to stress conditions and assess the viability of this spirochete, we used a correlative cryo-fluorescence and cryo-scanning microscopy approach. This approach enables simple exposition of bacteria to various experimental conditions that can be stopped at certain time intervals by cryo-immobilization, examination of cell viability without necessity to maintain suitable culture conditions during viability assays, and visualization of structures in their native state at high magnification. We focused on rare and transient events e.g., the formation of round bodies and the presence of membranous blebs in spirochetes exposed to culture medium, host sera either without or with the bacteriolytic effect and water. We described all crucial steps of the workflow, particularly the influence of freeze-etching and accelerating voltage on the visualization of topography. With the help of newly designed cryo-transport device, we achieved greater reproducibility.

## Introduction

Certain spirochete genospecies belonging to the *Borrelia burgdorferi* sensu lato complex are causative agents of Lyme disease (LD) and are transmitted by hard ticks of the genus *Ixodes* (Barbour and Hayes, 1986). *Borrelia* consist of a protoplasmic cell cylinder surrounded by an outer membrane and a plasma membrane with a peptidoglycan layer (Barbour and Hayes, 1986). Both membranes enclose a periplasmic space in which the flagella are located (Goldstein et al., 1994). Flagella define the flat-wave morphology of spirochetes and are responsible for their motility. Motility is a crucial factor in *Borrelia* transmission and its efficient dissemination through host/vector tissues (Motaleb et al., 2000). Mutations in the major flagella protein B result in the development of rod-shaped and non-motile spirochetes (Sultan et al., 2013). In addition to the flat-wave and rod-shaped morphological forms, the existence of non-motile atypical morphologies, such as looped/ring-shaped forms or spherical forms, has been previously described (Barbour and Hayes, 1986). Spherical cells are named in various ways, e.g., round bodies (RBs), spheroplast L-forms, cell wall-deficient, or cystic forms, and are described as large spherical structures with numerous flagella enclosed by an intact outer membrane (Hulínská et al., 1989, 1994; Mursic et al., 1996; Brorson et al., 2001; Miklossy et al., 2008; for reviews see Stricker and Johnson, 2011; Berndtson, 2013; Lantos et al., 2014). The transformation of motile *B. burgdorferi* spirochetes into non-motile RBs has been demonstrated *in vitro* in response to a hostile environment, e.g., after incubation with water, serum starvation or antibiotic treatment (Brorson and Brorson, 1997, 1998a; Alban et al., 2000; Murgia and Cinco, 2004; Brorson et al., 2009). Recently, Drecktrah et al. (2015) showed that during starvation, morphological conversion to RBs was dependent on the production of guanosine tetraphosphate and guanosine pentaphosphate. The transition of RBs back into the motile forms has been described after a short exposure of spirochetes to hypotonic conditions (Meriläinen et al., 2015). Next, spherical forms that were isolated from the spinal fluid converted back to the spiral form after cultivation in rich BSK-H medium (Brorson and Brorson, 1998b). Gruntar and colleagues showed the infectivity of *B. garinii* cystic forms prepared in dH_2_O that were intraperitoneally inoculated into mice (Gruntar et al., 2001). Cystic forms have been found in the brains of patients with neuropathologically confirmed Lyme neuroborreliosis (Miklossy et al., 2008), as well as in skin tissues (Aberer et al., 1996). Non-motile spirochetes have been visualized *in vivo* within the midgut of unfed *Ixodes scapularis* nymphs (Dunham-Ems et al., 2009).

Here, we present results from the first *in vitro* study on the formation and viability of atypical morphologic forms of infectious spirochetes *B. burgdorferi* s.s. expressing green fluorescent protein (GFP) in response to water and after incubation with sera. We used either sera from impala (*Aepyceros melampus*) or African buffalo (*Syncerus cifer*), that were determined in our earlier work to exert different bacteriolytic activities against spirochetes (Tichá et al., 2016). Impala serum revealed the strongest borreliacidal effect against all tested spirochete species from the *B. burgdorferi* s.l. complex in the complement sensitivity test, whereas buffalo serum did not display any borreliacidal effect (Tichá et al., 2016).

Cryo-fluorescence is a powerful imaging technique used for the visualization of fluorophore-tagged molecules in the frozen-hydrated state that opens new possibilities for correlative light electron microscopy studies (CLEM) at cryogenic temperatures (Chang et al., 2014; Kaufmann et al., 2014a,b; Schorb and Briggs, 2014; Strnad et al., 2015). Main advantages are derived from the cryo-immobilization and visualization of vitrified specimens without the influence of any chemicals and presence of artifacts caused e.g., during dehydration and drying steps included in the conventional sample preparation (Vancová and Nebesářová, 2015). Next, the cryo-fluorescence can be (re-)evaluated after cryo-SEM observations if needed, even after freeze-etching and gold sputter coating (Strnad et al., 2015). Another benefit of the cryo-fluorescence described earlier is its substantial reduction of photo-bleaching at low temperatures (Schwartz et al., 2007; Li et al., 2015). However, the crucial factors are maintaining the temperature of samples below −140°C to avoid formation of crystalline ice and prevent contamination. Proper vitrification of bulk specimens is another limiting factor for observation in cryo-SEM. Biological cryo-specimens are highly sensitive to electron-beam radiation damage, therefore electron beam exposures must be minimized and observation at very low energy (e.g., 1 kV) enable collection only “topographic” surface information (Nebesářová et al., 2016).

## Materials and methods

### *Borrelia* strain and culture conditions

*B. burgdorferi* Bb 914, a GFP-expressing virulent derivative of strain 297, was cultured in Barbour-Stoenner-Kelly medium (BSK-H, Sigma-Aldrich) containing 6% rabbit serum at 34°C until mid-log phase (~5 × 10^7^ spirochetes/ml). The GFP-tagged strain *B. burgdorferi* was obtained from Dr. Caimano (Caimano et al., 2004).

### Serum

Serum samples from male African buffalo (*Syncerus cifer*) and female impala (*Aepyrecoros melampus*) were obtained from the zoo in Hradec Kralove (Czech Republic; Tichá et al., 2016). Blood samples were taken during routine checks of the animals, following zoo ethics regulations.

### Cell preparation and viability staining

**Figure 1:**
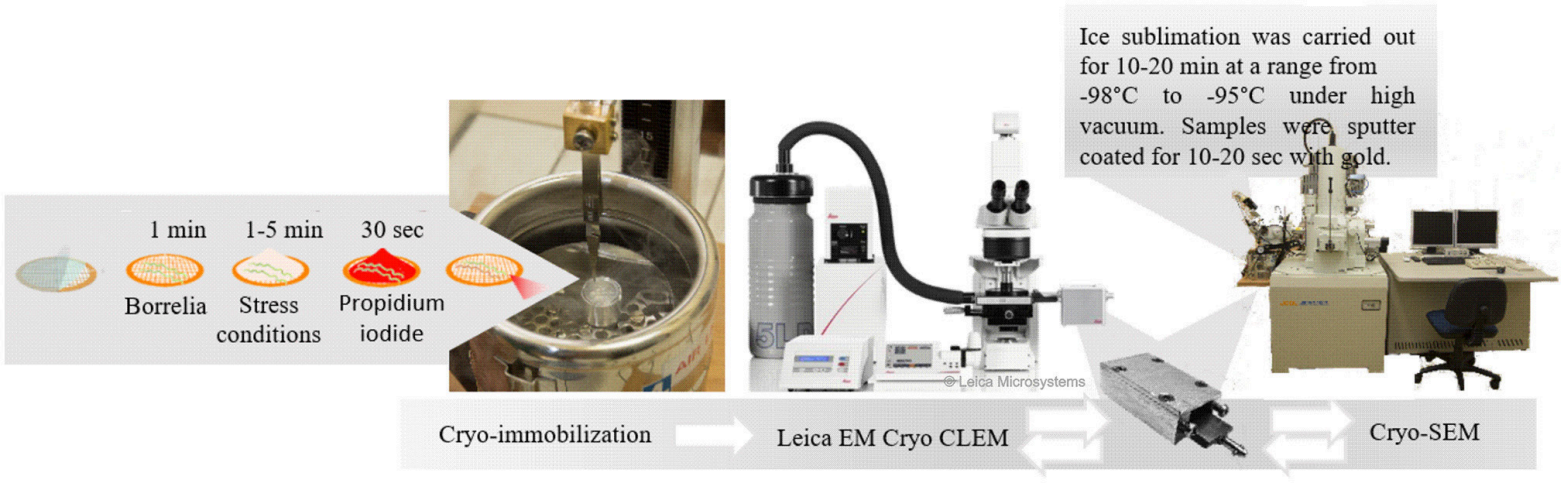
**Schematic overview of the workflow**. Copyright from Leica Microsystems.

A summary of the experimental approach is provided in Figure 1. Pelleted cells (7.5 × 10^7^) were washed in 0.1 M HEPES, centrifuged (820 × g, 10 min), resuspended in 100 μl of buffer, and immediately transferred onto freshly prepared 200-mesh index grids (e.g., Tedpella, Agar Scientific) coated with formvar-carbon film and glow discharged. After 1 min, either 3 μl of serum or culture media or dH_2_O was added. After incubation (1–5 min) at room temperature in a humidification chamber, 2 μl (1 μg/ml) of propidium iodide (PI, BioRad, λ excitation = 536 nm; λ emission = 620 nm) was added. After 30 s, excess solution was immediately removed with a piece of filter paper. Control cells were incubated in the presence of either water or BSK-H medium under the same conditions as described above.

### Cryo-fluorescence and cryo-scanning electron microscopy

**Figure 2:**
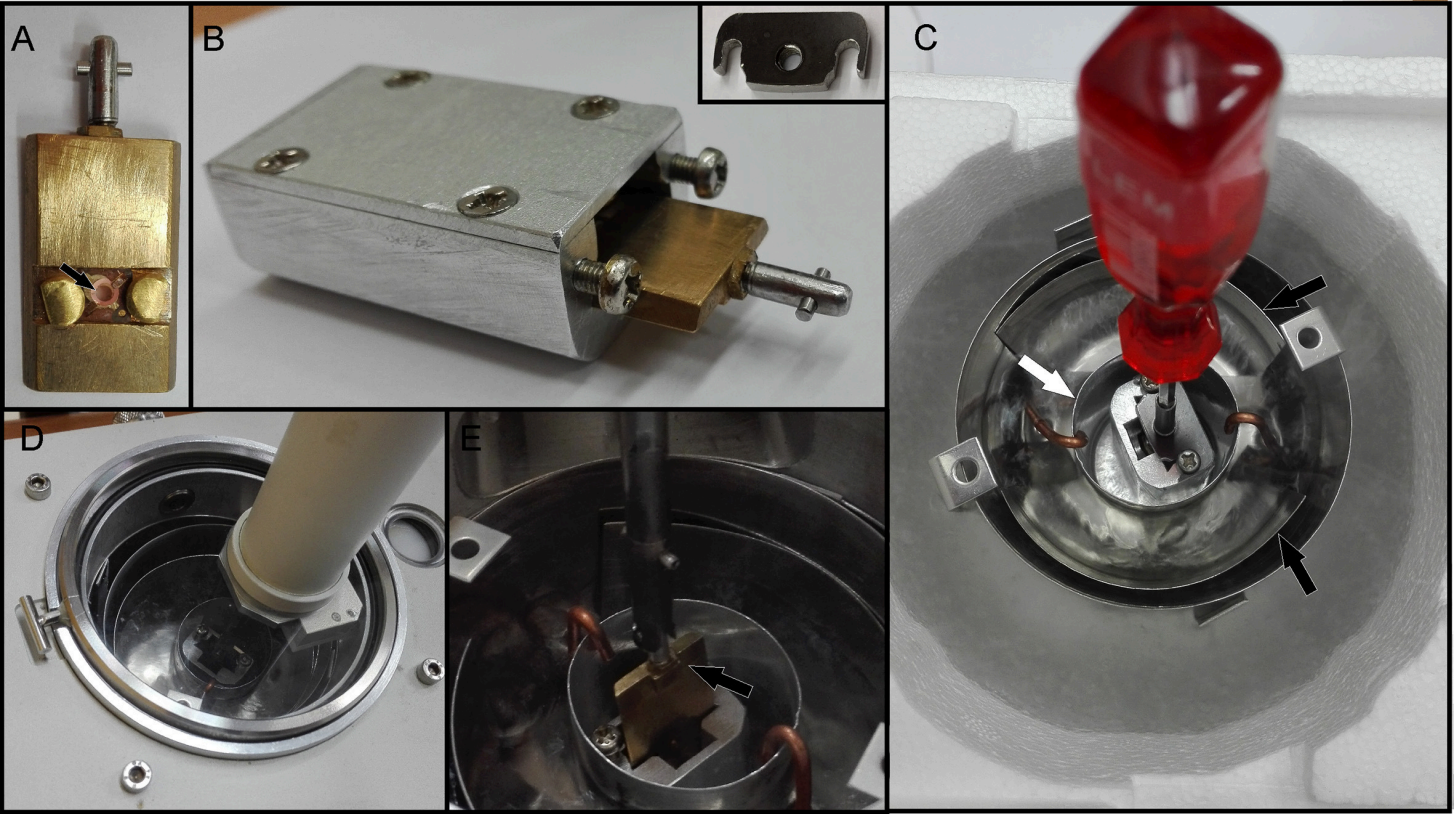
**Cryo-transfer of grids in nitrogen gas**. **(A)** The cartridge with the grid (arrow) is mounted onto a pre-cooled specimen cryo-holder (Gatan) and fixed using the tightening system. **(B)** The holder is inserted into the metal block and capped with a lid (inset) that is fixed with screws. **(C)** The homemade stainless steel cup is placed into a polystyrene box. The interior of the cup is divided by metal into several spaces (black arrows) and filled with liquid nitrogen. The central inner cup (white arrow) is cooled down only using nitrogen gas. When the liquid nitrogen stops boiling, the metal block is transferred into the inner cup. **(D)** The entire steel cup is transported to the CryoALTO slushing chamber. **(E)** The cryo-holder (arrow) containing the sample is transferred under vacuum to the chamber of the SEM.

**Figure 3:**
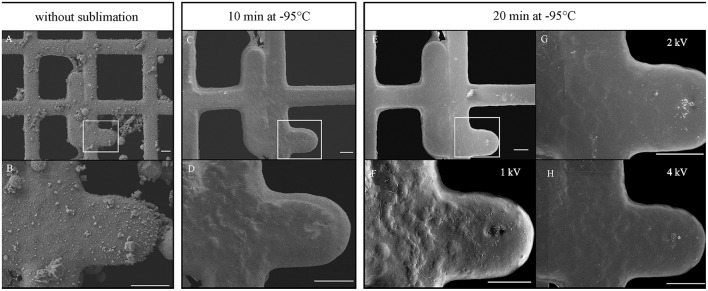
**Influence of ice sublimation time and accelerating voltage on the visualization of surface topography of spirochetes**. The same areas were imaged without sublimation **(A,B)** or after 10 min **(C,D)** and 20 min **(E–H)** of sublimation. Sputter-coated specimen was imaged with secondary electrons at 1 kV **(E,F)**, 2 kV **(G)** and 4 kV **(H)**. Images **(A–E)**, 1 kV. Bars 10 μm.

Grids were immediately frozen by plunging into liquid ethane cooled by liquid nitrogen using a homemade plunger. The excess of liquid ethane was blotted with filter paper and transferred to a Leica EM Cryo CLEM (Leica Microsystems, Vienna, Austria) system, comprising a shuttle device, a fluorescent light microscope (Leica DM6000 FS), a cryo CLEM objective (Leica HCX PL APO 50x/0.9), and a camera (Leica DFC310FX). Viability was determined by counting the cells using cryo-fluorescence. For topography, grids were transferred to a FESEM JEOL 7401F (JEOL Ltd.) equipped with a cryo-attachment (CryoALTO 2500, Gatan, Inc.). All transfers were performed strictly in the presence of liquid nitrogen vapors to minimize changes in temperature, ice contamination and cell loss. Inside the cryo-transfer shuttle (a part of the Leica EM Cryo CLEM system), the cartridge with the mounted grid (Figure 2A, arrow) was transferred to a pre-cooled standard specimen cryo-holder (Gatan, Inc.) adjusted for the secure transfer of the cartridge (Figure 2A). The holder was then inserted into a cavity of a pre-cooled homemade metal block and covered with a lid (Figure 2B, inset). The block was transferred from the Leica cryo-transfer shuttle into the pre-cooled homemade stainless steel cup placed in a polystyrene box (Figure 2C). The interior of the cup comprised areas separated by metal walls (Figure 2C, black arrow) that were filled with liquid nitrogen and a small central cup (Figure 2C, white arrow) for the transport of the metal block in nitrogen gas. The stainless cup with the metal block was transported to a CryoALTO slushing chamber (Figure 2D), where the cap was removed, and the chamber was evaporated. Finally, the holder was transported under vacuum (Figure 2E) to the preparation chamber using a transfer rod. Here, ice contaminants caused by the condensation of water vapors (Figures 3A,B) were removed by a 10-min sublimation at a range from −98°C to −95°C under high vacuum (Figures 3C,D). However, the surface topography of frozen spirochetes embedded into the thin layer of either serum or water was revealed after an additional 10-min sublimation under the same conditions (Figures 3E–H). Despite the extensive sublimation of the ice, neither specimen damage nor the presence of holes in the structure was observed. Next, the specimen surface was sputter-coated for 10–20 s with gold (cold sputter coater is a part of CryoALTO 2500), and images were recorded using the conventional Everhart-Thornley (ET) detector of secondary electrons at 1–3 kV at −140°C (Figures 3F–H). A graded increase in magnification enabled navigation to the area of interest. Fluorescence and SEM images were aligned using Adobe Photoshop.

## Results

### Morphological transformation and viability of spirochetes after incubation with sera

**Figure 4:**
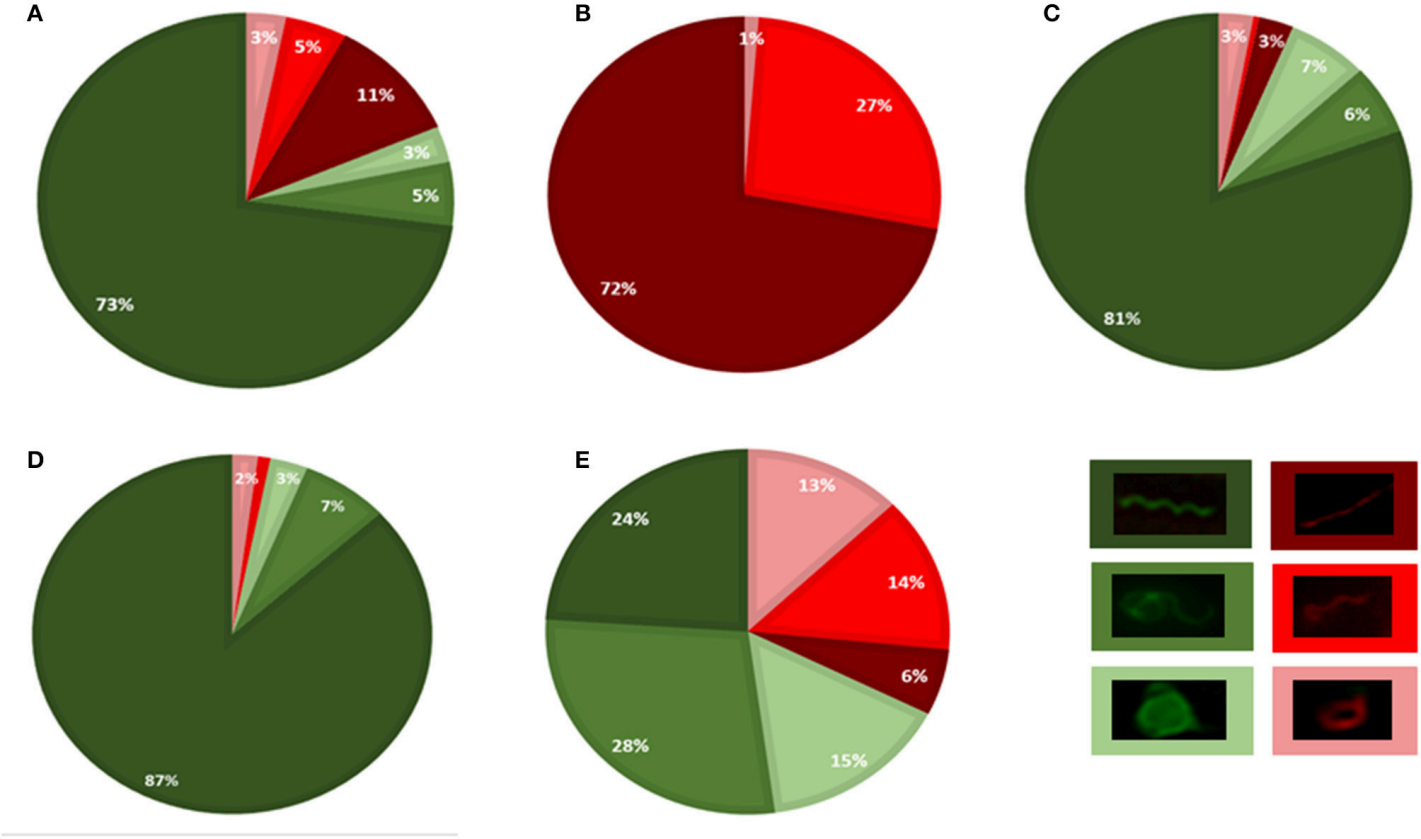
**GFP/PI viability assay performed by counting bacteria using Leica Cryo CLEM fluorescence microscopy**. *Borrelia* were incubated under different conditions: serum isolated from impala for 2 min **(A)** or 5 min **(B)**, or from African buffalo for 5 min **(C)**; BSK-H supplemented with 6% rabbit serum for 5 min **(D)** or with H_2_O for 2 min **(E)**. Three morphological variants were distinguished: spiral forms (dark green/red), irregularly shaped spirochetes (medium light green/red) and round body forms (light green/red). Viable bacteria (green), PI-stained bacteria (red).

**Figure 5:**
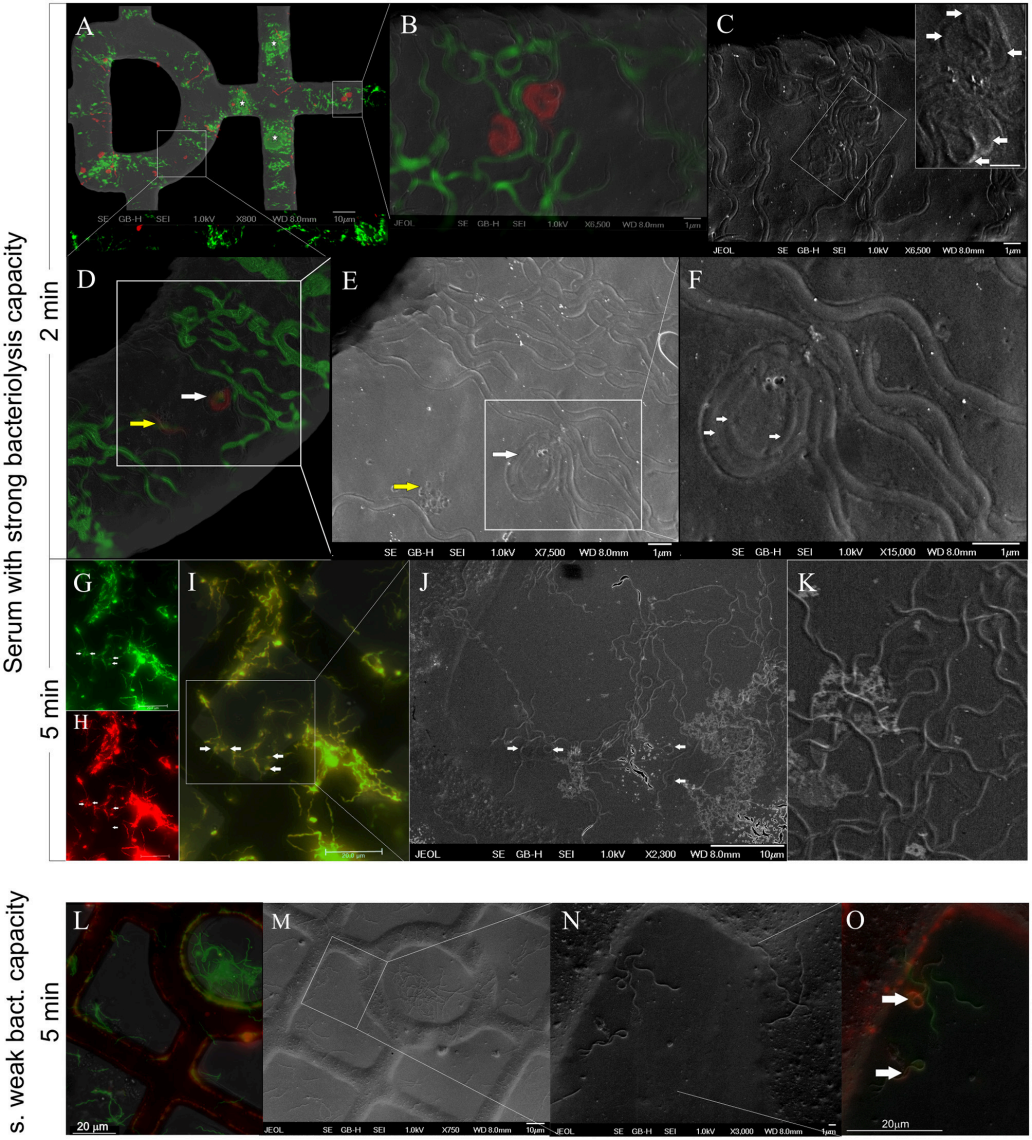
**GFP-expressing *B. burgdorferi* spirochetes incubated for 2 min (A–F)** or 5 min **(G–O)** in the presence of serum showing the strong **(A–K)** or weak **(L–O)** bacteriolysis capacity. Animal sera were isolated either from *Aepyceros melampus* **(A–K)** or *Syncerus cifer* **(L–O)**. After incubation, spirochetes were stained with propidium iodide (PI, red). **(A–F)** Cryo-fluorescence and cryo–SEM images merged with viability staining results and with the structural information of spirochetes at a different magnification. Areas overloaded by a high number of bacteria (**A**, asterisk). PI-stained damaged/dead bacteria in the form of the round body **(B,D)** and with a spiral-shaped morphology **(D,E)**. Regular (**F**, arrows) or irregular (**C**, inset-arrows) coils of the round body forms. Leakage of cellular content from the red-stained spirochete (**E**, yellow arrow). **G-K:** Flat-wave-shaped spirochetes with a frequent presence of blebs (**G–I**, arrows) that represent coiled ends/irregular parts of spirochetes as observed at high magnification **(J,K)**. **(L–O)** Representative image at low **(L,M)** and high **(N,O)** magnification. Irregularly shaped PI-positive parts of cells (**O**-arrows) shown on a merged secondary electron image and a fluorescence image.

After a 2-min incubation with impala serum, the majority of spirochetes were viable (81%, 237 cells) and had a flat-wave appearance (73%, 212 cells, Figure 4A). Other viable morphological forms, irregularly shaped spirochetes (5%, 16 cells) and viable RBs (3%, 9 cells) were observed rarely. Non-viable cells (19%, 54 cells, Figure 4A) had either a regular flat-wave shape (or were stretched, 11%, 31 cells), or an irregular cell morphology, e.g., coiled parts (5 %, 14 cells), and 3% represented red-stained RBs (9 cells; Figure 5). RB structures were formed by either three irregular or three regular folds of one bacterium (Figure 5, white arrows). A representative image shows a PI-stained spirochete with a flat-wave morphology (Figures 5D, yellow arrow) with membranous blebs or leaking cell content (Figure 5E, yellow arrow). An overload of several areas with a high amount of *Borrelia* prevented the recognition of individual cells (Figure 5A, asterisks). After the incubation time was extended to 5 min, all spirochetes were positively stained with PI (Figure 4B) and were spiral-shaped (72%, 238 cells), although straight spirochetes and bacteria with coiled ends (27%, 89 cells) and, rarely, RBs (1%, 4 cells) were observed (Figures 5G–K).

**Figure 6:**
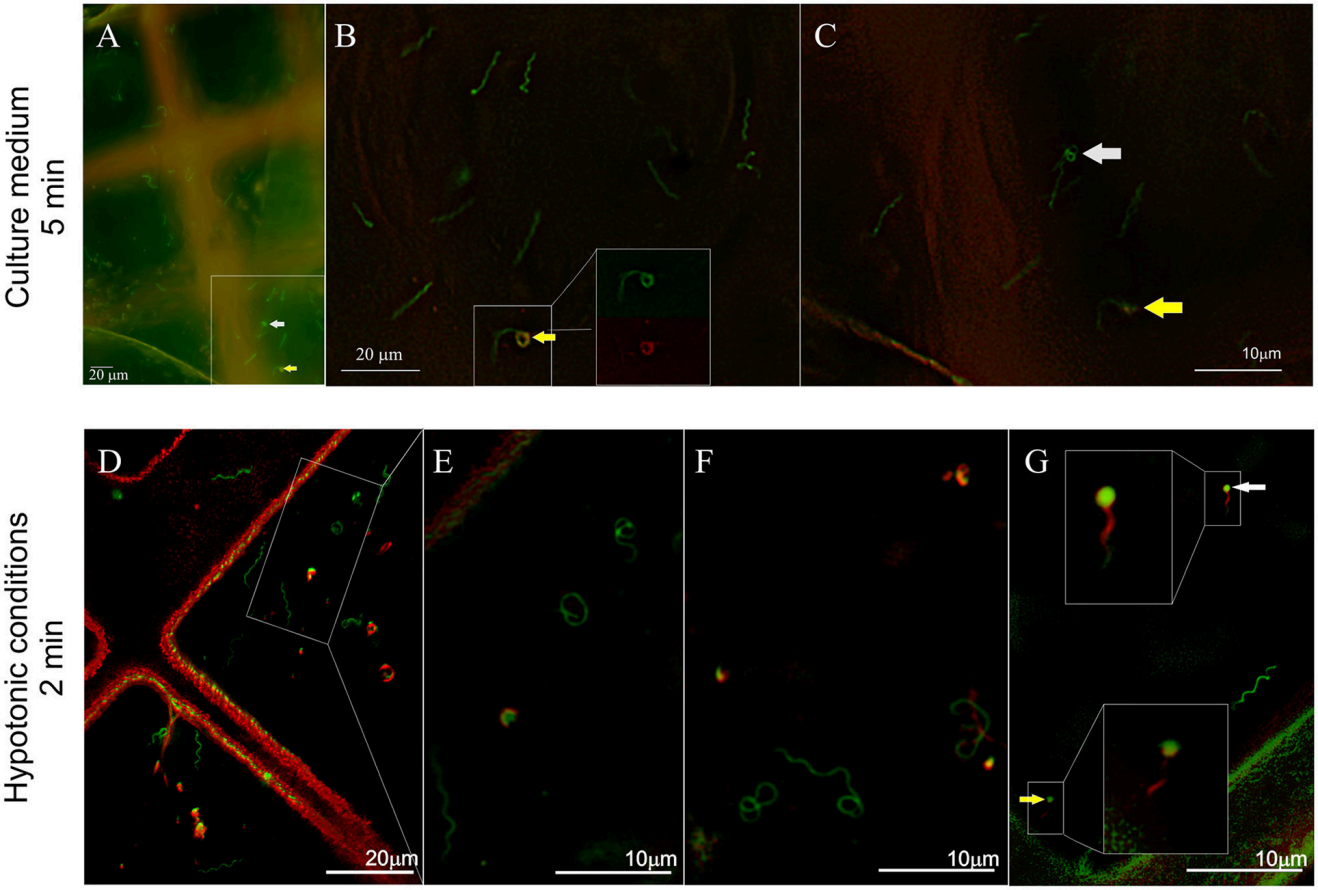
**Influence of hypotonic conditions on the morphology and viability of spirochetes expressing GFP**. Spirochetes were stained with propidium iodide (PI, red) and immediately frozen. **(A–C)** Majority of spirochetes incubated in culture medium for 5 min were flat-wave-shaped. Rarely, spirochetes with coiled ends (**B**, arrows) were observed (red and green fluorescence signals are shown in the inset). In another place, the coiled parts were PI negative (**C**, white arrow). **(D–G)** Significant morphological changes were observed after a 2-min incubation in presence of dH_2_O. Spirochetes partially stained with PI are shown in **G** (details in the insets).

In contrast to the impala serum, the majority of spirochetes incubated with African buffalo serum for 5 min were viable and had a flat-wave shape (73%, 169 cells, Figure 4C). Rarely, irregularly shaped forms (4%, 9 cells) or RB forms (3%, 8 cells) were found. PI-stained spirochetes (Figure 4C) exhibited either spiral (11%, 25 cells), irregular (6%, 13 cells) or RB forms (3%, 7 cells). Spirochetes with coiled ends were defined among irregularly shaped/PI-positive cells (Figures 5L–O). Similarly, the majority of spirochetes incubated in the presence of culture media (supplemented with 6% rabbit serum) for 5 min were viable (97%, 95 cells) and had a flat-wave morphology (87%). Individual cells were also irregular (7%) and in the form of RBs (3%). We rarely found spirochetes with atypical coiled ends that were counterstained with PI (Figures 6A–C).

### Morphological transformation and viability of spirochetes after short exposure to osmotic stress

Spirochetes exposed to short hypotonic stress for 1–3 min showed significant pleomorphism (Figures 4E, 6D,A–G). Only 24% (45 cells) of all viable cells (68%, total 127 cells) had a spiral morphology (Figure 4E), whereas 28% of spirochetes were irregularly shaped (53 cells), and 15% were spherical (29 cells). Cell viability was affected in 32% of cells (61 cells) that were either in the spiral (5.8%), irregular (13.8%), or RB forms (12.7%). These numbers also included spirochetes that were partially PI-positive, e.g., PI-stained spiral-shaped parts of the protoplasmic cylinders with green centers of coiled parts, as shown in Figure 6G (details shown in the insets).

## Discussion

We used a cryo-fluorescence and cryo-SEM approach for the rapid and close-to-native assessment of both the viability and morphological changes of GFP-expressing spirochetes exposed to physiological stress. The SEM approach allows the reliable differentiation of pleomorphic forms of *Borrelia* from other artifacts (e.g., background signals and unbound PI dye) and the visualization of the spatial organization of pleomorphic variants. Viable cells were distinguished from dead cells based on cell membrane integrity and PI permeability. This combination of green fluorescence emitted by cellular GFP and red fluorescence emitted by PI has been used previously to measure bacterial viability and has several advantages: the short amount of time required to perform the experiment, highly reliable results and no competition for binding sites in contrast to SYTO9/PI staining (Lehtinen et al., 2004). We found PI-positive cells that were either simultaneously GFP-positive (spirochetes incubated in the presence of impala serum for 5 min, Figures 5G–K) or only partially positive (control cells, spirochetes incubated in the presence of serum from African buffalo or water for 5 min, Figures 5L–O, 6D–G). A similar observation, in which a proportion of the dead GFP-tagged strain of *Pseudomonas* retained some GFP at a lower concentration, has been previously described (Lowder et al., 2000). We believe that this observation could be caused by a combination of different factors: (a) the extent of damage to the cell membrane, (b) the time of cell death/damage and/or (c) the difference in the molecule sizes of both GFP (27 kDa, the shape of a cylinder with a length of 4.2 nm and a diameter of 2.4 nm) and PI (0.668 kDa). The mechanism of cell death induced by hypo-osmotic shock probably involves cell burst after the extensive absorption of water and later leakage of GFP proteins. In contrast, during complement-mediated killing, terminal complement components form pores in the membranes, leading to damage, and death of the pathogen (Kurtenbach et al., 1998). The membrane-attack complex is ~10 nm in diameter (Janeway et al., 2001), which may explain the lower release of GFP from dying spirochetes after incubation with impala serum.

The influence of serum on the morphology of LD spirochetes has been previously described by de Taeye and Meriläinen (de Taeye et al., 2013; Meriläinen et al., 2015). RBs and blebs (large membrane bulges) were induced *in vitro* after incubation with culture media supplemented with human serum (de Taeye et al., 2013; Meriläinen et al., 2015). However, blebs (defined as small outer membrane vesicles) are released from microbial cell surfaces as a general response to stress (e.g., after antibiotic treatment, prolonged cultivation, addition of specific antibodies, and complement to culture) and are referred to as an initial sign of membrane alteration (Barbour and Hayes, 1986; de Taeye et al., 2013). We demonstrated here that in the presence of host sera (with and without a bacteriolytic effect), *B. burgdorferi* spirochetes do not change their typical flat-wave shape, even though RBs and spirochetes with bulges (which were often identified at high magnification as damaged coiling parts of the protoplasmic cylinder) were observed (Figure 5). We repeatedly observed low amount of viable and non-viable RBs forms (3%) in the control cultures and similarly after short exposure to host sera. In contrast, various viable pleomorphic forms of LD spirochetes developed after short exposure to osmotic shock; 68% of cells were still viable after 2 min; we observed 15% of viable and 13% of non-viable RBs (Figure 4). These results are consistent with the previous studies of Meriläinen and colleagues (Meriläinen et al., 2015), who showed that 85% of RBs were formed after a 10-min incubation with H_2_O and that spirochetes later reverted to their parent vegetative form after incubation in culture media.

These round morphological variants likely represent stressed viable bacteria that can revert, in some circumstances, back to the motile spiral form. The presence of atypical morphological forms of LD spirochetes *in vivo* and their direct association with chronic persistent infection in human or animal models has been reported in a small number of studies (Hulínská et al., 1989, 1994; Brorson et al., 2001; Miklossy et al., 2008). However, the significance of RBs in LD pathogenesis remains unclear (Lantos et al., 2014). The formation of different spirochetal forms can hypothetically explain the persistence of spirochete infection or the presence of unusual symptoms lasting for several months despite antibiotic treatment (Kersten et al., 1995; Golovchenko et al., 2016; Rudenko et al., 2016; Sapi et al., 2016). Recently, different bactericidal drugs targeting RBs and biofilm-like forms were tested *in vitro*, however, further *in vivo* studies are needed to evaluate their significance for treatment of “chronic” LD (Feng et al., 2016a,b,c). Similarly, findings on the viability of various morphological forms (e.g., RBs, blebs) of spirochetes can provide valuable insight into their role in the LD. For that purpose, we used (for the first time) a combination of cryo-fluorescence and cryo-scanning electron microscopy. In contrast to standard viability studies at ambient (or culture) temperature, the cryogenic conditions allowed us to freeze all dynamic events in the certain period, to stop the mobility of spirochetes, and to observe their morphology without influence of chemicals or unfavorable conditions that could be potential sources of artifacts. Cryo-fluorescence microscope Leica EM cryo CLEM, equipped with a HCX PL APO 50x objective with NA 0.9, is now commercially available and, in combination with cryo-EM, is becoming a powerful tool for imaging biological specimens.

## Author contributions

MV carried out the experiments; JV optimized the cryo-transfer and carried out SEM observations; MG and LT prepared the sera; MS contributed to protocol optimization, RR cultivated the GFP-*Borrelia*; JN and LG analyzed the data; MV, NR, and RR wrote the paper.

## Funding

This work was supported by the Technology Agency of the Czech Republic (TE01020118), European FP7 project ANTIGONE (278976), MEYS Czech Republic (Czech BioImaging LM2015062 and CZ.02.1.01/0.0/0.0/16_013/0001775). MS was supported by the Grant Agency of the University of South Bohemia (026/2015/P).

### Conflict of interest statement

The authors declare that the research was conducted in the absence of any commercial or financial relationships that could be construed as a potential conflict of interest.

## References

[B1] Aberer E. Kersten A. Klade H. Poitschek C. Jurecka W. (1996). Heterogeneity of *Borrelia burgdorferi* in skin. Am. J. Dermatopathol. 18, 571–579. 10.1097/00000372-199612000-00004 8989928

[B2] Alban P. S. Johnson P. W. Nelson D. R. (2000). Serum-starvation-induced changes in protein synthesis and morphology of *Borrelia burgdorferi*. Microbiology 146, 119–127. 10.1099/00221287-146-1-119 10658658

[B3] Barbour A. G. Hayes S. F. (1986). Biology of Borrelia species. Microbiol. Rev. 50, 381–400. 3540570 10.1128/mr.50.4.381-400.1986 PMC373079

[B4] Berndtson K. (2013). Review of evidence for immune evasion and persistent infection in Lyme disease. Int. J. Gen. Med. 6, 291–306. 10.2147/IJGM.S44114 23637552 PMC3636972

[B5] Brorson Ø. Brorson S. H. (1997). Transformation of cystic forms of *Borrelia burgdorferi* to normal, mobile spirochetes. Infection 25, 240–246. 10.1007/BF01713153 9266264

[B6] Brorson Ø. Brorson S. H. (1998a). A rapid method for generating cystic forms of *Borrelia burgdorferi*, and their reversal to mobile spirochetes. APMIS 106, 1131–1141. 10.1111/j.1699-0463.1998.tb00269.x 10052721

[B7] Brorson Ø. Brorson S. H. (1998b). *In vitro* conversion of *Borrelia burgdorferi* to cystic forms in spinal fluid, and transformation to mobile spirochetes by incubation in BSK-H medium. Infection 26, 144–150. 10.1007/BF02771839 9646104

[B8] Brorson O. Brorson S. H. Henriksen T. H. Skogen P. R. Schoyen R. (2001). Association between multiple sclerosis and cystic structures in cerebrospinal fluid. Infection 29, 315–319. 10.1007/s15010-001-9144-y 11787831

[B9] Brorson Ø. Brorson S. H. Scythes J. MacAllister J. Wier A. Margulis L. (2009). Destruction of spirochete *Borrelia burgdorferi* round-body propagules (RBs) by the antibiotic tigecycline. Proc. Natl. Acad. Sci. U.S.A. 106, 18656–18661. 10.1073/pnas.0908236106 19843691 PMC2774030

[B10] Caimano M. J. Eggers C. H. Hazlett K. R. O. Radolf J. D. (2004). RpoS is not central to the general stress response in *Borrelia burgdorferi* but does control expression of one or more essential virulence determinants. Infect. Immun. 72, 6433–6445. 10.1128/IAI.72.11.6433-6445.2004 15501774 PMC523033

[B11] Chang Y. W. Chen S. Tocheva E. I. Treuner-Lange A. Löbach S. Søgaard-Andersen L.. (2014). Correlated cryogenic photoactivated localization microscopy and cryo-electron tomography. Nat. Methods 11, 737–739. 10.1038/nmeth.2961 24813625 PMC4081473

[B12] de Taeye S. W. Kreuk L. van Dam A. P. Hovius J. W. Schuijt T. J. (2013). Complement evasion by *Borrelia burgdorferi*: it takes three to tango. Trends Parasitol. 29, 119–128. 10.1016/j.pt.2012.12.001 23298533

[B13] Drecktrah D. Lybecker M. Popitsch N. Rescheneder P. Hall L. S. Samuels D. S. (2015). Correction: The *Borrelia burgdorferi* RelA/SpoT homolog and stringent response regulate survival in the tick vector and global gene expression during starvation. PLoS Pathog. 11:e1005242. 10.1371/journal.ppat.1005242 26474045 PMC4608782

[B14] Dunham-Ems S. M. Caimano M. J. Pal U. Wolgemuth C. W. Eggers C. H. Balic A.. (2009). Live imaging reveals a biphasic mode of dissemination of *Borrelia burgdorferi* within ticks. J. Clin. Invest. 119, 3652–3665. 10.1172/JCI39401 19920352 PMC2786795

[B15] Feng J. Zhang S. Shi W. Zhang Y. (2016a). Ceftriaxone pulse dosing fails to eradicate biofilm-like microcolony *Borrelia burgdorferi* persisters which are sterilized by daptomycin/ doxycycline/cefuroxime without pulse dosing. Front. Microbiol. 7:1744 10.3389/fmicb.2016.01744 27867375 PMC5095124

[B16] Feng J. Shi W. Zhang S. Sullivan D. Auwaerter P. G. Zhang Y. (2016b). A drug combination screen identifies drugs active against amoxicillin-induced round bodies of *in vitro Borrelia burgdorferi* persisters from an FDA drug library. Front. Microbiol. 7:743. 10.3389/fmicb.2016.00743 27242757 PMC4876775

[B17] Feng J. Weitner M. Shi W. Zhang S. Zhang Y. (2016c). Eradication of biofilm-like microcolony structures of *Borrelia burgdorferi* by daunomycin and daptomycin but not mitomycin C in combination with doxycycline and cefuroxime. Front. Microbiol. 7:62 10.3389/fmicb.2016.00062 26903956 PMC4748043

[B18] Goldstein S. F. Charon N. W. Kreiling J. A. (1994). *Borrelia burgdorferi* swims with a planar waveform similar to that of eukaryotic flagella. Proc. Natl. Acad. Sci. U.S.A. 91, 3433–3437. 10.1073/pnas.91.8.3433 8159765 PMC43591

[B19] Golovchenko M. Vancová M. Clark K. Oliver J. H. Jr. Grubhoffer L. Rudenko N. (2016). A divergent spirochete strain isolated from a resident of the southeastern United States was identified by multilocus sequence typing as *Borrelia bissettii*. Parasit Vectors 9:68. 10.1186/s13071-016-1353-4 26846867 PMC4743114

[B20] Gruntar I. Malovrh T. Murgia R. Cinco M. (2001). Conversion of *Borrelia garinii* cystic forms to motile spirochetes *in vivo*. APMIS 109, 383–388. 10.1034/j.1600-0463.2001.090507.x 11478686

[B21] Hulínská D. Barták P. Hercogová J. Hancil J. Basta J. Schramlova J. (1994). Electron microscopy of Langerhans cells and *Borrelia burgdorferi* in Lyme disease patients. Zentralbl. Bakteriol. 280, 348–359. 10.1016/S0934-8840(11)80597-9 8167429

[B22] Hulínská D. Jirous J. Valesova M. Herzogova J. (1989). Ultrastructure of *Borrelia burgdorferi* in tissues of patients with Lyme disease. J. Basic Microbiol. 29, 73–83. 10.1002/jobm.3620290203 2709313

[B23] Janeway C. A. Jr. Travers P. Walport M. Shlomchik M. J. (eds.). (2001). The complement system and innate immunity, in Immunobiology: The Immune System in Health and Disease. 5th Edn. (New York, NY: Garland Science).

[B24] Kaufmann R. Hagen C. Grünewald K. (2014b). Fluorescence cryo-microscopy: current challenges and prospects. Curr. Opin. Chem. Biol. 20, 86–91. 10.1016/j.cbpa.2014.05.007 24951858 PMC4094034

[B25] Kaufmann R. Schellenberger P. Seiradake E. Dobbie I. M. Jones E. Y. Davis I.. (2014a). Super-resolution microscopy using standard fluorescent proteins in intact cells under cryo-conditions. Nano Lett. 14, 4171–4175. 10.1021/nl501870p 24884378 PMC4092024

[B26] Kersten A. Poitschek C. Rauch S. Aberer E. (1995). Effects of penicillin, ceftriaxone, and doxycycline on morphology of *Borrelia burgdorferi*. Antimicrob. Agents Chemother. 39, 1127–1133. 10.1128/AAC.39.5.1127 7625800 PMC162695

[B27] Kurtenbach K. Sewell H. S. Ogden N. H. Randolph S. E. Nuttall P. A. (1998). Serum complement sensitivity as a key factor in Lyme disease ecology. Infect Immun. 66, 1248–1251. 9488421 10.1128/iai.66.3.1248-1251.1998 PMC108041

[B28] Lantos P. M. Auwaerter P. G. Wormser G. P. (2014). A systematic review of *Borrelia burgdorferi* morphologic variants does not support a role in chronic Lyme disease. Clin. Infect Dis. 58, 663–671. 10.1093/cid/cit810 24336823 PMC3922218

[B29] Lehtinen J. Nuutila J. Lilius E. M. (2004). Green fluorescent protein–propidium iodide (GFP-PI) based assay for flow cytometric measurement of bacterial viability. Cytometry A 60A, 165–172. 10.1002/cyto.a.20026 15290717

[B30] Li W. Stein S. C. Gregor I. Enderlein J. (2015). Ultra-stable and versatile widefield cryo-fluorescence microscope for single-molecule localization with sub-nanometer accuracy. Opt. Express 23, 3770–3783. 10.1364/OE.23.003770 25836229

[B31] Lowder M. Unge A. Maraha N. Jansson J. K. Swiggett J. Oliver J. D. (2000). Effect of starvation and the viable-but-nonculturable state on green fluorescent protein (GFP) fluorescence in GFP-tagged *Pseudomonas fluorescens* A506. Appl. Environ. Microbiol. 66, 3160–3165. 10.1128/AEM.66.8.3160-3165.2000 10919764 PMC92128

[B32] Meriläinen L. Herranen A. Schwarzbach A. Gilbert L. (2015). Morphological and biochemical features of *Borrelia burgdorferi* pleomorphic forms. Microbiology 161, 516–527. 10.1099/mic.0.000027 25564498 PMC4339653

[B33] Miklossy J. Kasas S. Zurn A. D. McCall S. Yu S. McGeer P. L. (2008). Persisting atypical and cystic forms of *Borrelia burgdorferi* and local inflammation in Lyme borreliosis. J. Neuroinflammation 5:40 10.1186/1742-2094-5-40 18817547 PMC2564911

[B34] Motaleb M. A. Corum L. Bono J. L. Elias A. F. Rosa P. Samuels D. S.. (2000). *Borrelia burgdorferi* periplasmic flagella have both skeletal and motility functions. Proc. Natl. Acad. Sci. U.S.A. 97, 10899–10904. 10.1073/pnas.200221797 10995478 PMC27121

[B35] Murgia R. Cinco M. (2004). Induction of cystic forms by different stress conditions in *Borrelia burgdorferi*. APMIS 112, 57–62. 10.1111/j.1600-0463.2004.apm1120110.x 14961976

[B36] Mursic V. P. Wanner G. Reinhardt S. Wilske B. Busch U. Marget W. (1996). Formation and cultivation of *Borrelia burgdorferi* spheroplast-L-form variants. Infection 24, 218–226. 10.1007/BF01781096 8811359

[B37] Nebesářová J. Wandrol P. Vancová M. (2016). Novel method of simultaneous multiple immunogold localization on resin sections in high resolution scanning electron microscopy. Nanomedicine 12, 105–108. 10.1016/j.nano.2015.09.008 26472050

[B38] Rudenko N. Golovchenko M. Vancova M. Clark K. Grubhoffer L. Oliver J. H. (2016). Isolation of live *Borrelia burgdorferi* sensu lato spirochaetes from patients with undefined disorders and symptoms not typical for *Lyme borreliosis*. Clin. Microbiol. Infect 22, e9–e15. 10.1016/j.cmi.2015.11.009 26673735

[B39] Sapi E. Balasubramanian K. Poruri A. Maghsoudlou J. S. Socarras K. M. Timmaraju A. V.. (2016). Evidence of *in vivo* existence of *Borrelia biofilm* in borrelial lymphocytomas. Eur. J. Microbiol. Immunol. 6, 9–24. 10.1556/1886.2015.00049 27141311 PMC4838982

[B40] Schorb M. Briggs J. A. (2014). Correlated cryo-fluorescence and cryo-electron microscopy with high spatial precision and improved sensitivity. Ultramicroscopy 143, 24–32. 10.1016/j.ultramic.2013.10.015 24275379 PMC5472196

[B41] Schwartz C. L. Sarbash V. I. Ataullakhanov F. I. McIntosh J. R. Nicastro D. (2007). Cryo-fluorescence microscopy facilitates correlations between light and cryo-electron microscopy and reduces the rate of photobleaching. J. Microsc. 227, 98–109. 10.1111/j.1365-2818.2007.01794.x 17845705

[B42] Stricker R. B. Johnson L. (2011). Lyme disease: the next decade. Infect Drug Resist. 4, 1–9. 10.2147/IDR.S15653 21694904 PMC3108755

[B43] Strnad M. Elsterová J. Schrenková J. Vancová M. Rego R. O. M. Grubhoffer L.. (2015). Correlative cryo-fluorescence and cryo-scanning electron microscopy as a straightforward tool to study host-pathogen interactions. Sci. Rep. 5:e18029. 10.1038/srep18029 26658551 PMC4674872

[B44] Sultan S. Z. Manne A. Stewart P. E. Bestor A. Rosa P. A. Charon N. W.. (2013). Motility is crucial for the infectious life cycle of *Borrelia burgdorferi*. Infect. Immun. 81, 2012–2021. 10.1128/IAI.01228-12 23529620 PMC3676011

[B45] Tichá L. Golovchenko M. Oliver J. H. Jr. Grubhoffer L. Rudenko N. (2016). Sensitivity of Lyme borreliosis spirochetes to serum complement of regular zoo animals: potential reservoir competence of some exotic vertebrates. Vector Borne Zoonotic Dis. 16, 13–19. 10.1089/vbz.2015.1847 26783940

[B46] Vancová M. Nebesářová J. (2015). Correlative fluorescence and scanning electron microscopy of labelled core fucosylated glycans using cryosections mounted on carbon-patterned glass slides. PLoS ONE 10:e0145034. 10.1371/journal.pone.0145034. 26690057 PMC4699470

